# Interactions between the oomycete *Pythium arrhenomanes* and the rice root-knot nematode *Meloidogyne graminicola* in aerobic Asian rice varieties

**DOI:** 10.1186/s12284-016-0108-3

**Published:** 2016-07-29

**Authors:** R. E. M. Verbeek, C. G. B. Banaay, M. Sikder, D. De Waele, C. M. Vera Cruz, G. Gheysen, M. Höfte, Tina Kyndt

**Affiliations:** 1 Department of Molecular Biotechnology, Faculty of Bioscience Engineering, Ghent University, B-9000 Ghent, Belgium; 2 Laboratory of Phytopathology, Department of Crop Protection, Faculty of Bioscience Engineering, Ghent University, B-9000 Ghent, Belgium; 3 International Rice Research Institute, Los Baños, Laguna Philippines; 4 Department of Biosystems, Faculty of Bioscience Engineering, University of Leuven (KU Leuven), B-3001 Heverlee, Belgium; 5 Unit for Environmental Sciences and Management, North-West University, 2520 Potchefstroom, South Africa; 6 Current address: Institute of Biological Sciences, University of the Philippines, Los Baños, Laguna Philippines; 7 Current address: Laboratory of Mycology and Plant Pathology, Department of Botany, Jahangirnagar University, Savar, Dhaka, 1342 Bangladesh

**Keywords:** Antagonism, Nematode development, Aerobic rice

## Abstract

**Background:**

Aerobic rice fields are frequently infested by pathogenic oomycetes (*Pythium* spp.) and the rice root-knot nematode *Meloidogyne graminicola*. Here, the interaction between *Pythium arrhenomanes* and *Meloidogyne graminicola* was studied in rice roots of two aerobic rice varieties. In different experimental set-ups and infection regimes, plant growth, rice yield, *Pythium* colonization, as well as establishment, development and reproduction of *M. graminicola* were studied.

**Results:**

In this study, it is shown that the presence of *P. arrhenomanes* delays the establishment, development and reproduction of *M. graminicola* compared to single nematode infected plants. The delay in establishment and development of *M. graminicola* becomes stronger with higher *P. arrhenomanes* infection pressure.

**Conclusions:**

Our data indicate that *P. arrhenomanes* antagonizes *M. graminicola* in the rice root and that the plant benefits from this antagonism as shown by the yield data, especially when either of the pathogens is present in high levels.

**Electronic supplementary material:**

The online version of this article (doi:10.1186/s12284-016-0108-3) contains supplementary material, which is available to authorized users.

## Background

Asian rice (*Oryza sativa* L.) is traditionally cultivated in paddy fields where the plants are grown anaerobically in a layer of water. This cultivation system uses a large quantity of the available water in rice-producing countries, especially in Asia (Peng et al. 2006). To utilize water more efficiently, rice farmers are increasingly adopting less water-consuming farm practices. The aerobic rice production system is considered one of the most promising adaptation strategies to grow more rice with less water and to react effectively to the looming water crisis (Tuong and Bouman 2003). In the last decennia, aerobic Asian rice varieties have been bred that can compete in yield with traditional rice varieties (Bouman et al. 2005; Lafitte et al. 2002; Sandhu et al. 2013). However, continuous cropping of aerobic rice in the same field resulted in yield decline (Peng et al. 2006), rapid yield losses (George et al. 2002), and even yield failure (Kreye et al. 2009b). The cause of these reductions in yield is still unclear but there are increasing indications that root pathogens that can build up large population densities on aerobic rice may be the most important factors affecting growth and yield of tropical aerobic rice (Peng et al. 2006; Kreye et al. 2009a; Kreye et al. 2009b). Common root pathogens found in aerobic rice fields are oomycetes (*Pythium* spp.) and the rice root-knot nematode *Meloidogyne graminicola* (Kreye et al. 2009b).

Plant pathogenic *Pythium* species are known to colonize seeds, seedlings and young plant tissues, causing pre- and post-emergence damping off. Infection is most often not lethal but may result in wilting and stunting of rice seedlings, and yield decline (Martin and Loper 1999). Van Buyten et al. (2013) isolated and identified three *Pythium* species (i.e., *P. inflatum, P. graminicola, and P. arrhenomanes*) associated with plant growth reduction of aerobic rice in the Philippines, of which *Pythium arrhenomanes* was shown to be the most virulent species. These *Pythium* species have been isolated from a wide range of economically important crops (including maize, barley, sorghum and sugarcane), but can also thrive on wild grasses and weeds (Van Buyten et al. 2013). Chun & Schneider (1998), studying the infection cycle of *Pythium* spp. on rice seeds and seedlings, noticed that the zoospores were selectively attracted towards germinating rice embryos and colonized the primary radicle. Plants infected with *Pythium* spp. three days after germination showed less stunting than earlier infected plants, indicating an increased resistance and/or tolerance in older plants. An in vitro study by Van Buyten & Höfte (2013) showed that hyphae of *P. arrhenomanes* grow intracellularly and colonize the cortical and endodermal cells within 27 h after infection. Subsequently, hyphae colonize the xylem, thus blocking water transportation to the shoot, resulting in stunting.

*Meloidogyne graminicola* is one of the most predominant nematode species associated with Asian rice. It has been found in every country in South and Southeast Asia surveyed so far (Soriano and Reversat 2003; De Waele et al. 2013; De Waele and Elsen 2007). *Meloidogyne graminicola* is a sedentary endoparasitic nematode and rice infection is characterized by hook-shaped galls (root-knots), mainly on the root tips (Kyndt et al. 2014). Under optimal conditions, the duration of its life cycle is 2 to 4 weeks at ambient temperatures of 25-35 °C (Fernandez et al. 2013; Plowright and Bridge 1990). After establishing a feeding site in the root vascular tissue, the infective second-stage juveniles (J2) molt three times to become mature, swollen females which lay eggs inside the roots (Kyndt et al. 2014). The feeding site consists of so-called giant cells surrounding the head of the female. These cells act as a metabolic sink to provide the female with nutrients. Parasitism by *M. graminicola* deforms the vascular tissue thus limiting water and nutrient transport and this may lead to reduced plant growth and lower yield (Padgham et al. 2004; Vovlas et al. 2005).

Under field conditions, a variety of pathogens may attack a crop. However, studies on interactions between these biotic stress factors are usually scarce (Atkinson and Urwin 2012). Although infections by *Meloidogyne* spp. and *Pythium* spp. frequently co-occur in rice fields, their interaction has not been studied. Nevertheless, interactions between *Pythium* species and plant-parasitic nematodes have been described on chrysanthemum (Johnson and Littrell 1970), tobacco (Khan and Haque 2013), and sugarcane (Bond et al. 2004). In sugarcane, *P. arrhenomanes* was able to suppress the reproduction of the ectoparasitic nematodes *Tylenchorhynchus annulatus* and *Mesocriconema xenoplax*, but not of *Paratrichodorus minor*. In contrast, *P. arrhenomanes* colonization was inhibited by high infection with a mixture of the three ectoparasitic nematodes (Bond et al. 2004). *Pythium aphanidermatum* was able to suppress the egg production of *Meloidogyne incognita*, but not of *Belonolaimus longicaudatus* in chrysanthemum. However, when both nematodes and the oomycete were present, plant disease became more severe compared to single infections showing a synergistic interaction between the oomycete and the two nematodes (Johnson and Littrell 1970). In tobacco, a *P. aphanidermatum* population in the soil became significantly larger in the presence of *M. incognita*, whereas the nematode population in soil decreased in the presence of the oomycete. In the roots a similar interaction was observed, where *P. aphanidermatum* reduced root galling compared to *M. incognita* alone and *M. incognita* accelerated the pathogenesis of *P. aphanidermatum*. However, the plant growth was more reduced when both pathogens were present (Khan and Haque 2013).

The objectives of our research were to (i) investigate if there is an interaction between *P. arrhenomanes* and *M. graminicola* in rice, (ii) study the population dynamics of both pathogens and their interaction in two different rice varieties throughout a rice crop cycle in the Philippines, and (iii) to examine the interaction between both pathogens using different inoculation schemes.

## Results

In a preliminary phytotron experiment, plant growth and yield was evaluated in single and double infected rice of the variety IR81413-BB-75-4 in comparison with un-inoculated plants (Additional file 1: Fig. S1). The plants in sterile soil were on average 20 % taller than the plants infected with either *Pythium arrhenomanes* or *Meloidogyne graminicola* alone or in combination (Additional file 1: Fig. S1A). At 17 days after germination (DAG), *P. arrhenomanes* infected plants were also significantly (*p* = 0.003) smaller compared to the single *M. graminicola* infected plants. However, this difference disappeared at later time points. At harvest, the grain weight of the plants infected only with *P. arrhenomanes* was similar to the grain weight of the control plants (Additional file 1: Fig. S1B), while plants infected with *M. graminicola* alone had a 47 % lower grain weight (*p* = 0.05) compared to the control plants. The grain weight of the plants infected both with *P. arrhenomanes* and *M. graminicola* was not significantly different from the grain weight of the control plants.

### Raised bed experiment

**Fig. 1:**
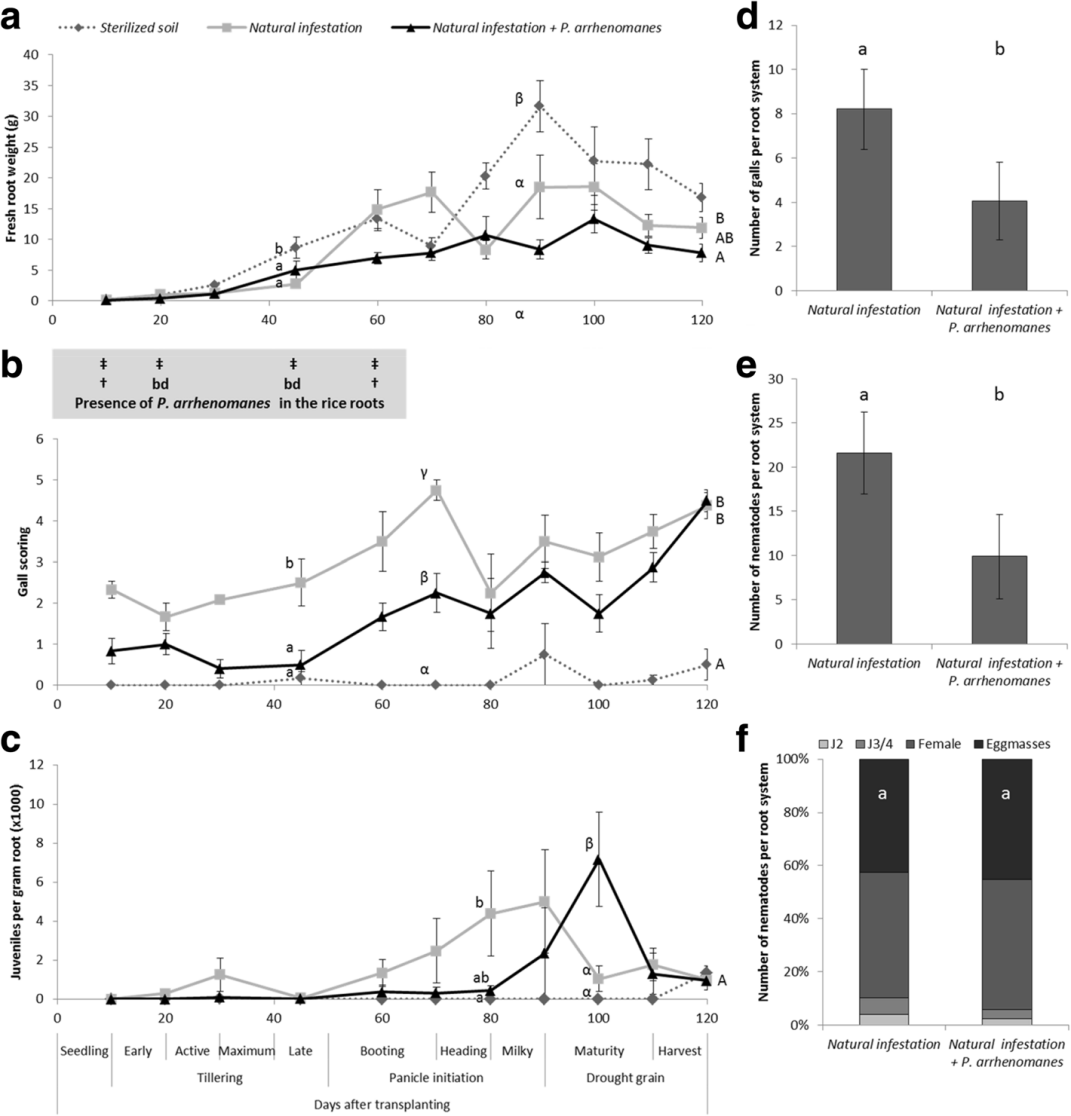
Growth variables of Palawan in raised bed. Sterilized soil = soil taken from field B912 and steamed; Natural infestation = soil taken from field B912 and; Natural infestation + *P. arrhenomanes* = B912 soil with additional *P. arrhenomanes* inoculation. **a** Fresh root weight over the cropping season. †‡ indicate the presence of *Pythium arrhenomanes* DNA in rice roots in naturally infested soil (bottom row) and naturally infested soil + *P. arrhenomanes,* (top row) respectively. bd indicates that the level of *Pythium arrhenomanes* DNA in rice roots is below detection. **b** Gall scoring on a scale of 5; 0 = 0 %, 1 = <10 %, 2 = 10-25 %, 3 = 26-50 %, 4 = 51-75 %, 5 = 75 < % of roots infected with galls. **c** Population dynamics of *Meloidogyne graminicola* juveniles in rice roots (*n* = 4 to 8). **d** Number of galls, **e** number of nematodes and **f** their developmental stages at 20 days after transplanting. Statistics were performed with One-way ANOVA Duncan test (α = 0.05; **a**, **b**, **c**) or Mann-Whitney *U* test (α = 0.05; **d**, **e**, **f**), different letters indicate significant differences. Error bars are the standard error (*n* = 4 to 8 for a, b, c; *n* = 15 for **d**, **e**, **f**)

**Fig. 2:**
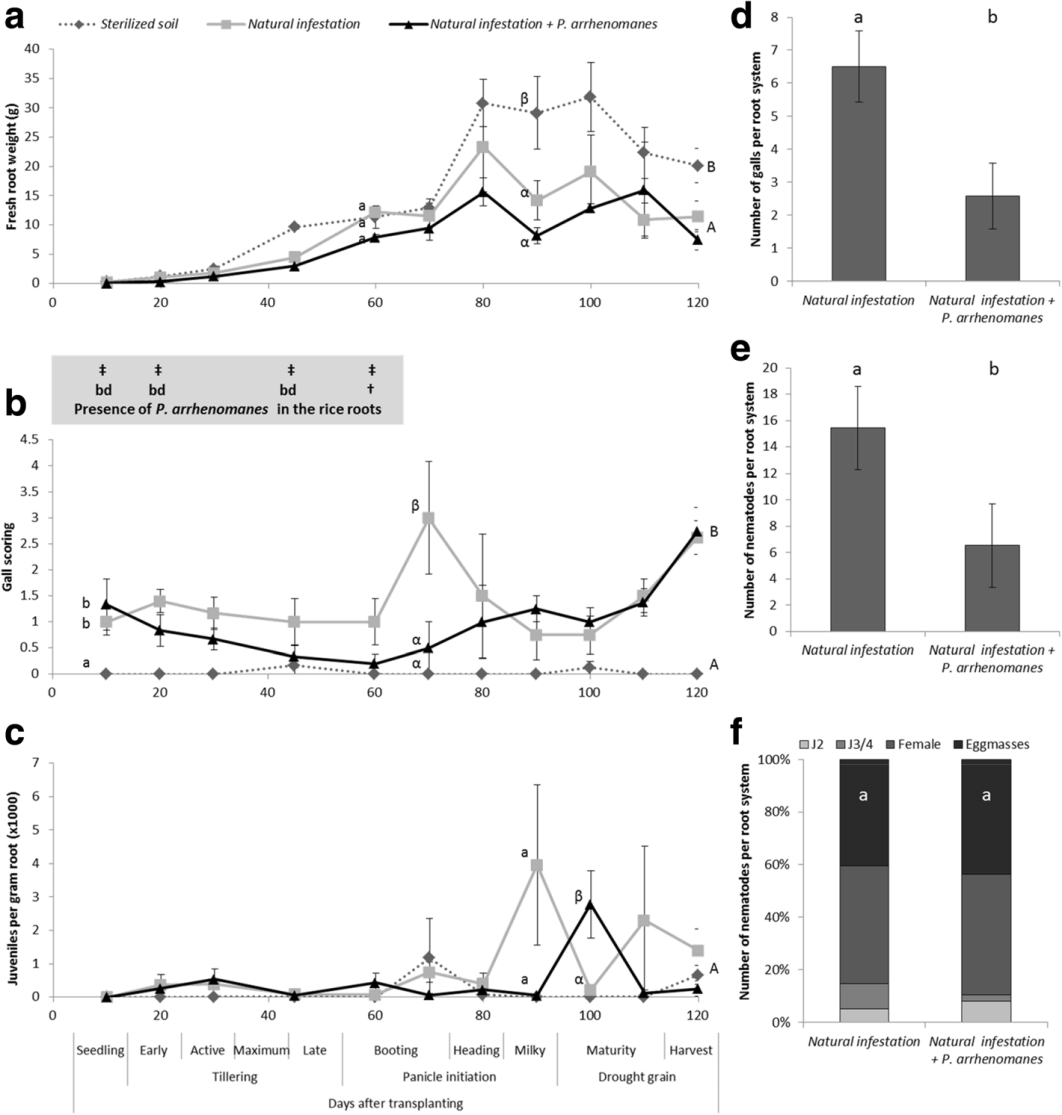
Growth variables of IR881413-BB-75-4 in raised bed. Sterilized soil = soil taken from field B912 and steamed; Natural infestation = soil taken from field B912 and; Natural infestation + *P. arrhenomanes* = B912 soil with additional *P. arrhenomanes* inoculation. **a** Fresh root weight over the cropping season. †‡ indicate the presence of *Pythium arrhenomanes* DNA in rice roots in naturally infested soil (bottom row) and naturally infested soil + *P. arrhenomanes,* (top row) respectively. bd indicates that the level of *Pythium arrhenomanes* DNA in rice roots is below detection. **b** Gall scoring on a scale of 5; 0 = 0 %, 1 = <10 %, 2 = 10-25 %, 3 = 26-50 %, 4 = 51-75 %, 5 = 75 < % of roots infected with galls. **c** Population dynamics of *Meloidogyne graminicola* juveniles in rice roots. **d** Number of galls, **e** number of nematodes and **f** their developmental stages at 20 days after transplanting. Statistics were performed with One-way ANOVA Duncan test (α = 0.05; **a**, **b**, **c**) or Mann-Whitney *U* test (α = 0.05; **d**, **e**, **f**), different letters indicate significant differences. Error bars are the standard error (*n* = 4 to 8 for **a**, **b**, **c**; *n* = 15 for **d**, **e**, **f**)

A raised bed experiment was set up to evaluate the population dynamics of *M. graminicola* and the presence of *P. arrhenomanes* in roots of two aerobic rice varieties, Palawan (Fig. 1) and IR81413-BB-75-4 (Fig. 2). In a first treatment, natural infection was used (Natural infestation), while a second treatment was additionally inoculated with extra *P. arrhenomanes* (Natural + *P. arrhenomanes*) to enhance its infection pressure. Control plants, grown in sterilized soil, had at several evaluated time points significantly higher root weight compared to the two infested treatments for both varieties (Figs. 1a & 2a), although this was not consistent throughout the growth season. Under natural infection pressure, *P. arrhenomanes* DNA could only be detected at 10 and 60 days after transplantation (DAT) in Palawan (Fig. 1a, Additional file 2: Figure S3A), and only at 60 DAT in IR81413-BB-75-4 (Fig. 2a, Additional file 2: Figure S3B). Under higher infection pressure, *P. arrhenomanes* could be detected at all measured time points (10, 20, 45 & 60 DAT; Additional file 2: Fig. S3A, B).

Population dynamics of *M. graminicola* was evaluated by gall scoring on the rice roots (Figs. 1b & 2b) and by extracting J2 from the roots during the crop cycle (Figs. 1c & 2c). For both rice varieties, a distinct peak in number of J2 per gram of root was observed in the naturally infested soil at the milky stage of plant development (90 DAT). This peak was delayed by 10 days in the raised beds where an additional *P. arrhenomanes* inoculation was performed. For both varieties a peak in gall scoring could be observed 20 days preceding the J2 peaks under naturally infested soil, while with an additional *P. arrhenomanes* inoculation the gall scoring peak preceded the J2 peaks with only 10 days.

The number of galls, nematodes and the developmental stages of *M. graminicola* were assessed at 20 DAT, after about one life cycle of *M. graminicola*. A significant (*p* ≤ 0.006) decrease in number of galls (Figs. 1d & 2d) and total number of nematodes (Figs. 1e & 2e) was observed in plants grown in soil with additional *P. arrhenomanes* inoculation, compared to plants grown in naturally infested soil. There was no shift in nematode development observed at this time point (Figs. 1f & 2f).

**Fig. 3:**
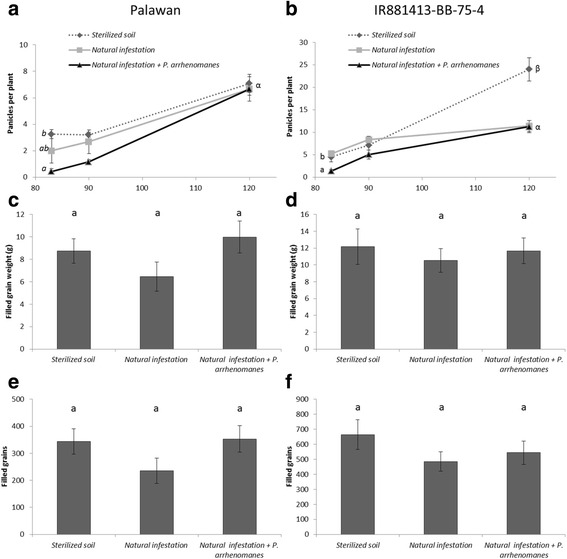
Yield data of raised bed experiment for Palawan **a**, **c**, **e** and IR81413-BB-75-4 **b**, **d**, **f**. Sterilized soil = soil taken from field B912 and steamed; Natural infestation = soil taken from field B912 and; Natural infestation + *P. arrhenomanes* = B912 soil with additional *P. arrhenomanes* inoculation. **a**, **b** Panicle emergence at 83, 90 and 120 days after germination. Four independent samples were taken (*n* = 4), each consisting of a pool of 30 plants for the first two time points, at harvest 12 plants were sampled (*n* = 12). **c**, **d** Filled grain weight per plant (*n* = 12). **e**, **f** Number of filled grains per plant (*n* = 12). Statistics were performed with Mann-Whitney *U* test (α = 0.05), different letters indicate significant differences. Error bars are the standard error

Panicle emergence started at 64 DAG in Palawan, and 2 weeks later in IR81413-BB-75-4. Figure 3 shows data of the panicle emergence of both varieties, recorded at 83, 90 DAG, and at harvest (120 DAG). At 83 and 90 DAG, the plants in the naturally infested soil showed the same panicle emergence pattern as plants grown in un-inoculated, sterilized soil. In contrast, for both varieties we observed a significant (*p* ≤ 0.021) delay in panicle emergence in the soil with high *P. arrhenomanes* infection pressure*,* where 7.5 (Palawan) and 3 times (IR81413-BB-75-4) less panicles were observed at 83 DAG compared to the control plants. This delay was still observed at 90 DAG in Palawan. However, at 120 DAG, no significant differences in the number of panicles were observed for Palawan. In contrast, for IR81413-BB-75-4, plants grown in sterilized soil had 2.2 times more panicles at harvest compared to plants grown in infested soils.

At harvest the filled grain weight (Fig. 3c,d) and number of filled grains (Fig. 3e, f) were evaluated. For Palawan, plants grown in naturally infested soils showed a reduction in grain weight compared to the plants grown in sterilized soil, although this effect was not significant (*p* = 0.057). This effect was however not observed with higher *Pythium* infection pressure (*p* = 0.644). For IR81413-BB-75-4, no difference in yield was observed between plants grown in sterilized soil and naturally infested soils (*p* ≥ 0.603).

### Greenhouse experiments

To confirm and extend the observations made in the phytotron and raised beds experiments, an experiment was performed under controlled conditions in a greenhouse. Here, several artificial inoculation treatments were compared; (i) un-inoculated control; (ii) *M. graminicola* inoculated; (iii) *P. arrhenomanes* inoculated; (iv) *P. arrhenomanes* + *M. graminicola*; (v) *M. graminicola* + *P. arrhenomanes* 6 days later; and (vi) *P. arrhenomanes* + *M. graminicola* 5 days later.

**Fig. 4:**
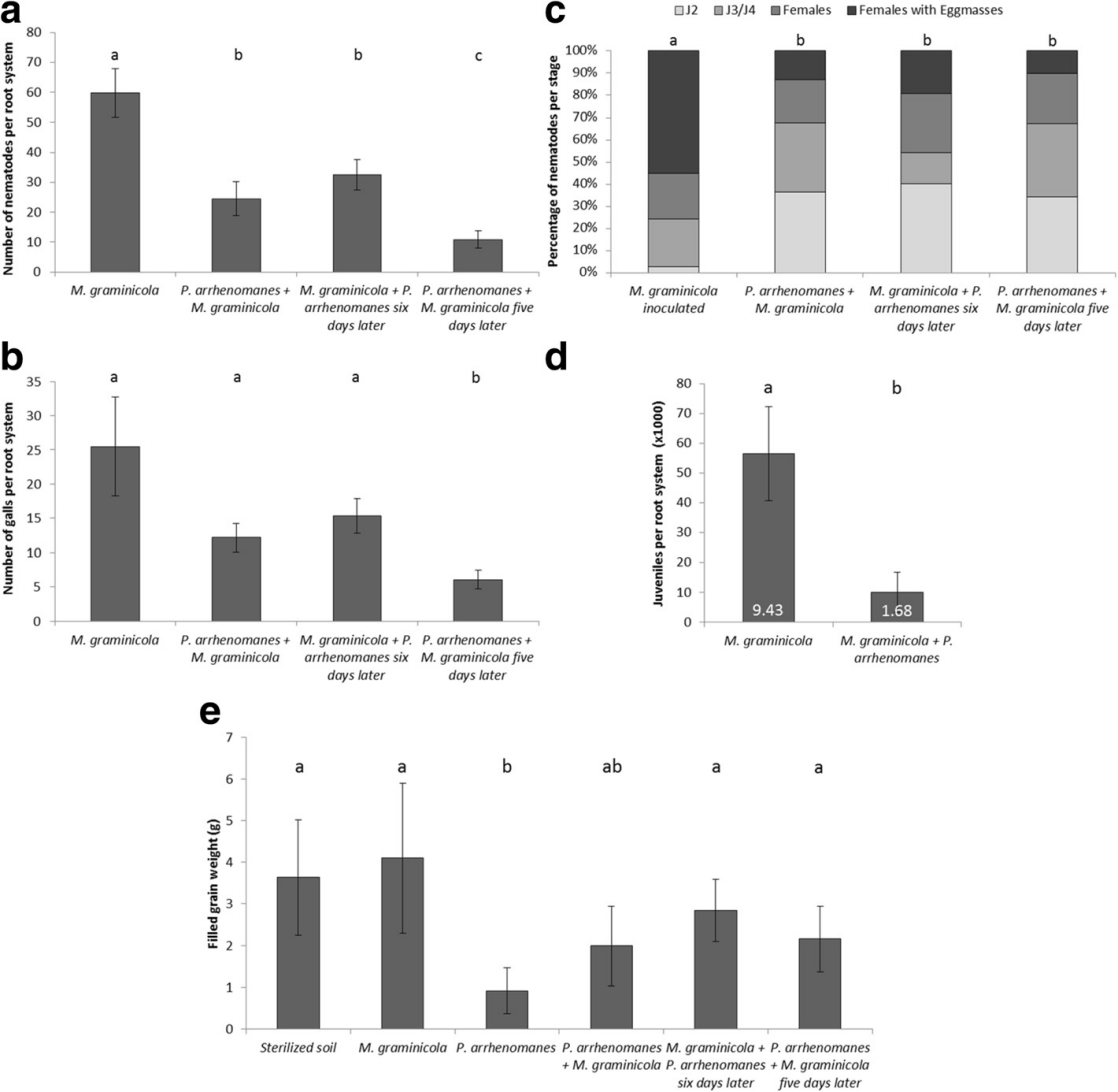
Nematode infection data and yield of Palawan in greenhouse under different infection schemes. **a** Total number of nematodes, **b** number of galls, and **c** the developmental stages of *M. graminicola* per plant at 15 days after infection. **d** Average number of juveniles extracted from rice roots at 54 days after infection from *M. graminicola* single infection and *M. graminicola* + *P. arrhenomanes* infection. The numbers in the bars are the Pf/Pi-values (Final population/ initial population). **e** Filled grain weight per plant (*n* = 12). Statistics were performed with Mann-Whitney *U* test (α = 0.05), different letters indicate significant differences (*n* = 12). Statistics for **c** were performed by giving each group a total score; where the percentage of each stage has a value; J2 = 1; J3/J4 = 2; Females = 3; and Females with Egg masses = 4. Error bars are the standard error

**Fig. 5:**
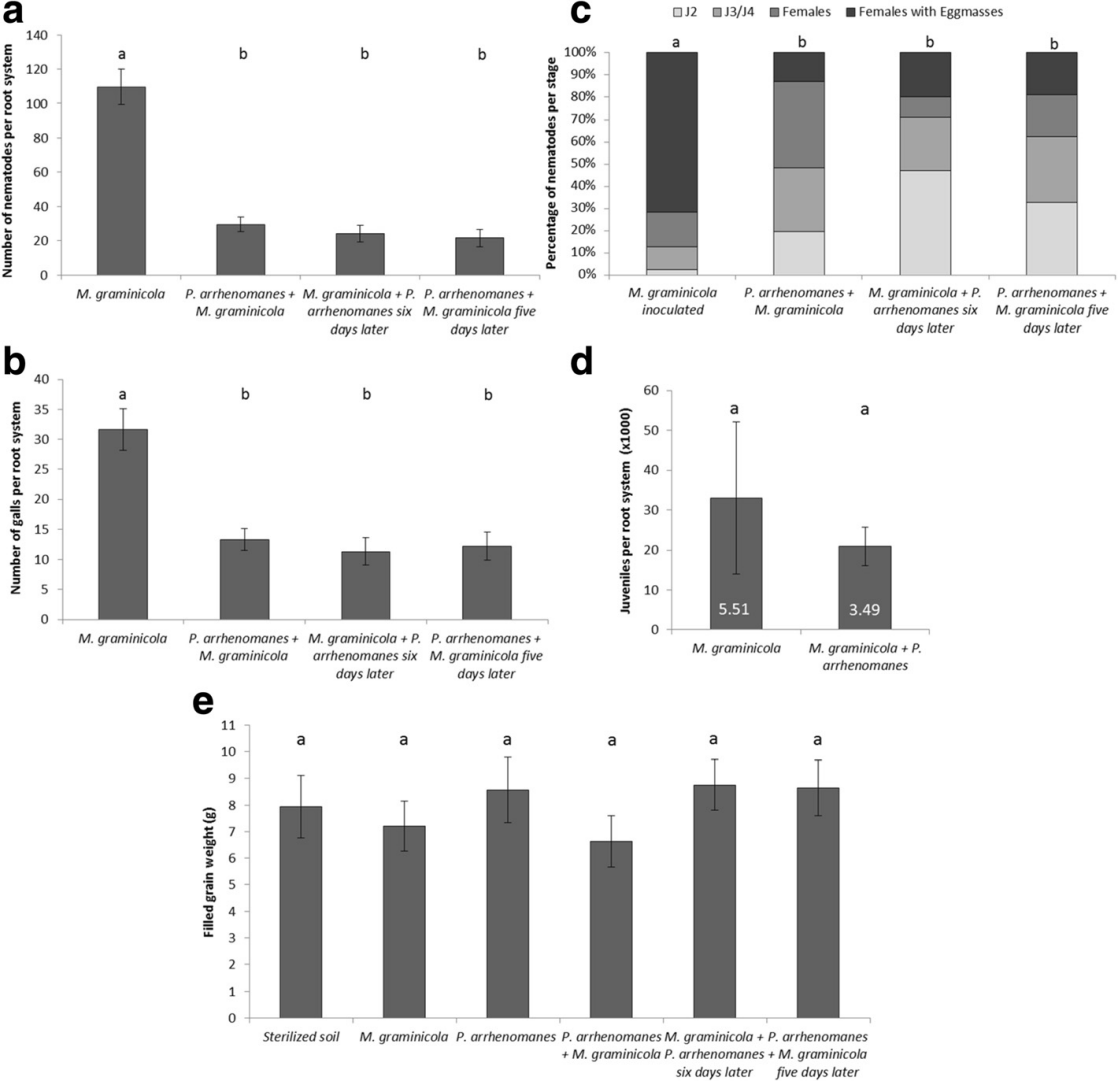
Nematode infection data and yield of IR881413-BB-75-4 in greenhouse under different infection schemes. **a** Total number of nematodes, **b** number of galls, and **c** the developmental stages of *M. graminicola* per plant at 15 days after infection. **d** Average number of juveniles extracted from rice roots at 54 days after infection from *M. graminicola* single infection and *M. graminicola* + *P. arrhenomanes* infection. The numbers in the bars are the Pf/Pi-values (Final population/ initial population). **e** Filled grain weight per plant (*n* = 12). Statistics were performed with Mann-Whitney *U* test (α = 0.05), different letters indicate significant differences (*n* = 12). Statistics for **c** were performed by giving each group a total score; where the percentage of each stage has a value; J2 = 1; J3/J4 = 2; Females = 3; and Females with Egg masses = 4. Error bars are the standard error

All treatments with *M. graminicola* were sampled at 15 and 20 days after infection (DAI) to assess the nematode development. For both rice varieties, the number of nematodes were significantly (*p* ≤ 0.029) lower in all treatments where *Pythium* was co-infected, in comparison with single nematode inoculation (Figs. 4a, 5a & Additional file 3: Figure S2A,B). The same observations were made for the number of galls (Figs. 4b, 5b & Additional file 3: Figure S2C,D), although there was no significance for Palawan at 15 DAI (Fig. 4b). Pre-, post- and simultaneous inoculation of *P. arrhenomanes* in respect to *M. graminicola* inoculation did not influence these observations. In both varieties, the single nematode infected roots show significantly more developed nematodes compared to the plants infected with both pathogens, regardless of the inoculation regime (Figs. 4c, 5c & Additional file 3: Figure S2E,F). The level of *P. arrhenomanes* colonization was similar between *P. arrhenomanes* single infected roots and double infected roots (Additional file 2: Fig. S3C).

At 54 DAI, which theoretically corresponds to the 3rd generation of *M. graminicola*, juveniles were extracted. The number of extracted juveniles and the corresponding Pf/Pi-values are shown in Figs. 4d & 5d. When comparing the single *M. graminicola* infected plants of both varieties, the reproduction rate of *M. graminicola* is about 70 % higher in Palawan compared to IR81413-BB-75-4. In Palawan, a significantly (*p* = 0.002) lower number of juveniles and a 5.5 times lower nematode reproduction rate was recorded from roots where both pathogens are present compared to single *M. graminicola* infected roots. In IR81413-BB-75-4 there is a similar trend, although the difference is not significant (*p* = 0.534).

As a measure of rice yield, the filled grain weight was evaluated at the end of the experiment (Figs. 4e & 5e). For IR81413-BB-75-4, the various inoculation regimes did not significantly influence the grain weight. Palawan plants infected with *M. graminicola* produced the same yield as the un-inoculated control plants. For Palawan, there was a significant (*p* = 0.008) reduction in filled grain weight after *P. arrhenomanes* single infection compared to the sterilized control. However, in combination with *M. graminicola, P. arrhenomanes* did not cause significant yield losses in this variety (*p* ≥ 0.129).

## Discussion

Numerous studies on rice pathogens have been done, but there have been few reports on root pathogens and their interactions. Here we report that *P. arrhenomanes* antagonize *M. graminicola* in the rice root, an antagonism from which the plant benefits.

When focusing on grain yield, the antagonistic effect of *Pythium* presence negatively affecting *M. graminicola* was seen most clearly in the preliminary phytotron experiment with variety IR81413-BB-75-4, where the grain yield was reduced after *M. graminicola* single infection, whereas plants infected with both pathogens showed a similar yield as the control plants. The reason why the antagonism was seen most clearly in this set-up is most likely due to the high infection pressure of both pathogens in this system. The direct seeding technique used in this experiment will promote *P. arrhenomanes* colonization since seeds and young seedlings are more susceptible to *P. arrhenomanes* compared to older seedlings. Within the first 4 days after germination rice seedlings become resistant to *Pythium spp.* (Chun and Schneider 1998; Halpin and Hanson 1958; Van Buyten 2013). Therefore it is likely that *P. arrhenomanes* was able to colonize the roots to a higher extent in the phytotron experiment and antagonized *M. graminicola* more effectively.

In support of this, our raised bed experiment confirmed that mainly under high infection pressure, *P. arrhenomanes* is able to reduce nematode establishment, root galling and delay nematode development. The population of *M. graminicola* showed a single peak in number of J2 at the milky stage of plant development (90 DAT), with a delay of 10 days when plants where grown under high *P. arrhenomanes* infection pressure. A previous population dynamics study on *M. graminicola* by Win et al. (2013) showed two distinctive peaks throughout the crop cycle, with a 1st peak at the maximum tillering stage and a 2nd peak around the heading stage. Both studies were done in different countries under different conditions with naturally infested soil, which makes it difficult to compare the data. The delaying effect of *P. arrhenomanes* on the *M. graminicola* development was confirmed under greenhouse conditions, where fewer galls and a delay in nematode development inside the roots were observed when both pathogens were present together. Similar observations were made while studying *Meloidogyne* spp. and *Pythium* spp. interactions on chrysanthemum (Johnson and Littrell 1970) and tobacco (Khan and Haque 2013). An interaction study with *P. arrhenomanes* and the migratory ectoparasitic nematode *Tylenchorhynchus annulatus* showed a significantly decreased reproduction rate of the nematode when co-inoculated with a high oomycete inoculum (Bond et al. 2004), revealing that this antagonism is not only limited to sedentary nematodes. However, no negative effect of *P. arrhenomanes* on reproduction of ectoparasitic nematode *Belonolaimus longicaudatus* was observed in chrysanthemum (Johnson and Littrell 1970).

Next to a delay in nematode development when *P. arrhenomanes* is present in the soil, we observed a general negative effect of *P. arrhenomanes* on nematode penetration and/or feeding site initiation. This might be caused by a potential negative influence of *P. arrhenomanes* on the attractiveness of the roots. Root attractiveness is mainly determined by root exudates (Bais et al. 2006). A study with two fungal species (i.e.*, Cochliobolus sativus* and *Fusarium culmorum*) showed that volatile organic compounds (VOCs) from the roots affected the growth of one fungus when barley roots were infected with the other fungus (Fiers et al. 2013). No data are currently available on alteration of the root exudates by *P. arrhenomanes* colonization, but this hypothesis deserves further investigation.

However, changes in root exudates might not be the only explanation for the observed antagonism. In the greenhouse experiment, we observed that the inoculation order of *P. arrhenomanes* and *M. graminicola* did not influence the level of antagonism. It was remarkable that, compared with single nematode inoculated plants, significantly less galls and nematodes were found in plants infected with *P. arrhenomanes*, even if the *P. arrhenomanes* inoculation was done 6 days after nematode inoculation. Under optimal conditions *M. graminicola* infects within the first days, with visible galls at 3 days after infection. These data indicate that either the plant is not able to provide enough nutrients to the nematodes to maintain a feeding site or that *P. arrhenomanes* might be able to disintegrate the feeding sites. This hypothesis is supported by histological studies of Melendez and Powell (1967), who showed that *Fusarium* can colonize nematode-induced giant cells. Since *Pythium* spp. are known to colonize the vascular tissue (Van Buyten and Höfte 2013; Yadeta and J. Thomma 2013), a colonization of the nematode feeding site might explain the here-reported antagonism.

An alternative explanation could be that the nematodes leave *P. arrhenomanes* colonized roots because the root’s nutritional capacity is weakened. A recent study by Ji et al*.* (2014) showed that rice roots treated with defense elicitor β-aminobutyric acid 2 days post *M. graminicola* infection, had slightly less nematodes inside the roots than non-treated plants. In addition, the number of *M. incognita* juveniles in the roots of resistant alfalfa decreased significantly at 8 days after infection compared to a susceptible alfalfa variety (Griffin and Elgin 1977). These reports and our data indicate that *Meloidogyne* spp. could emigrate from the roots when conditions are unfavorable even up to 8 days after infection. However, since this hypothesis contradicts with the general knowledge that juveniles of *Meloidogyne* spp. become sedentary at 2-3 days after rice root infection (Bridge et al. 2005), we believe that nematode emigration at 6 days after nematode inoculation is rather unlikely.

*M. graminicola* secretes a variety of enzymes in the giant cells that degrade cellulose, hemi-cellulose or pectin for easier digestion (Gheysen and Mitchum 2011). In cotton, it has been demonstrated that alterations in the xylem fluid caused by *M. incognita* infection lead to enhanced spore germination of *Fusarium* and *Verticillium* (Minton and Minton 1963). Similarly*, P. arrhenomanes* could potentially take advantage of the cellular degradation products released by nematode migration and feeding, resulting in fewer nutrients available for *M. graminicola,* hence leading to a slower nematode development. It should however be noted that a higher *P. arrhenomanes* density in the soil in the raised bed experiment did not delay the development of *M. graminicola* any further. Based on the fact that *Pythium* spp. infect only young plants and its presence is hard to detect beyond seedling stage (Chun and Schneider 1998), our analyses focused less on the potential of *M. graminicola* negatively affecting *P. arrhenomanes* colonization. However, yield parameters are useful parameters to evaluate *P. arrhenomanes* damage on rice plants. Yield data from the greenhouse experiment shows that Palawan suffered strongly from the single *P. arrhenomanes* infection, but this effect was generally less severe when both pathogens are present, indicating that *M. graminicola* also antagonizes *P. arrhenomanes* in rice roots. This could however not be confirmed by quantification of *P. arrhenomanes* DNA in the plant roots in the greenhouse experiment, were *P. arrhenomanes* DNA levels were similar between *P. arrhenomanes* single infected plants and *P. arrhenomanes* + *M. graminicola* double infected plants (Additional file 2: Figure S3C).

Quantitative PCR revealed that *P. arrhenomanes* DNA concentrations in the root system were generally below the detection limit in roots grown in naturally infested soil, whereas *P. arrhenomanes* DNA could be detected in most roots grown in soil with additional *P. arrhenomanes* colonization (Additional file 2: Fig. S3A,B). The level of *P. arrhenomanes* DNA in the roots has been studied previously in vitro by Van Buyten et al*.* (2013), who showed that *P. arrhenomanes* levels reached between 179 to 590 pg/ng total DNA within 3 days of infection, causing severe stunting and seedling death at 10 days after infection. In our study the *P. arrhenomanes* DNA levels ranged from 100 to 300 pg/ng total DNA (Additional file 2: Fig. S3), but seedling death could not be observed. The fact that *P. arrhenomanes* did not have any visual effect on the rice seedlings might be due to the transplanting of 7 day old seedlings. Chun and Schneider (1998) studied pathogenicity of *Pythium* species in rice seedlings and described an increased resistance of rice seedlings towards *Pythium* species starting 4 days after germination. In older plants, root colonization by *Pythium* spp. has not been described. However, here we demonstrate that *P. arrhenomanes* is able to colonize the roots of 7 days-old rice plants under field conditions, and that *P. arrhenomanes* DNA is detectable in the root system up to 60 days after transplanting, while only causing yield losses in Palawan in the greenhouse experiment. In the field, *Pythium* species were re-isolated from rice roots up to 68-77 days after sowing, with positive ITS identification for *P. arrhenomanes* at 37 days after sowing (personal communication; Banaay, G.). This shows that *P. arrhenomanes* remains viable in mature plants.

From our experiments it can be concluded that both varieties are susceptible to both pathogens, as *P. arrhenomanes* could be detected in the roots of both varieties in the raised bed experiment and high nematode population densities and severe root galling by *M. graminicola* was observed in all experiments. Severe root galling is usually related with high yield loss (personal communication; De Waele, D.), but based on the yield data of the greenhouse experiment it can be concluded that both varieties are tolerant to *M. graminicola*. The tolerance of Palawan to *M. graminicola* observed in the greenhouse experiment contradicts the results of a study by De Waele et al. (2013), where Palawan was shown to be sensitive to *M. graminicola*. Tolerance of rice varieties towards *M. graminicola* is difficult to confirm, as it depends on many factors (personal communications; De Waele, D.). Tolerance towards *P. arrhenomanes* also depends on different factors, as Palawan showed to be sensitive to *P. arrhenomanes* in the greenhouse experiment, but in the raised bed experiment a high *P. arrhenomanes* pressure gave a similar filled grain weight as un-inoculated plants.

## Conclusions

Overall the here-reported experiments show that *P. arrhenomanes* antagonizes *M. graminicola* in the rice root system. The underlying mechanisms are however still unclear and molecular, biochemical and histopathological techniques are required to give more insight. In practice it might be interesting to introduce *P. arrhenomanes* into the soil when the seedlings are more mature and hence resistant to *Pythium*, as a strategy to prevent nematode damage on nematode susceptible rice varieties. *P. arrhenomanes* is still able to enter the roots, but will not cause any reduction on grain yield, if the variety has a certain tolerance to *P. arrhenomanes*. Our data indicates that in variety Palawan *M. graminicola* can alleviate yield losses caused by *P. arrhenomanes* infection. On the other hand, in both varieties *P. arrhenomanes* can suppress nematode establishment, development and reproduction, ultimately reducing yield losses caused by *M. graminicola.*

## Methods

### Preparation of pathogen inoculum

*Pythium arrhenomanes* (PT60), isolated from an aerobic rice field in Tarlac, Philippines (Van Buyten et al. 2013), was maintained in water agar plugs submerged in sterile distilled water and kept at 15 °C. Working cultures were revived on potato dextrose agar (PDA) and incubated at 28 °C. Final inocula were prepared by inoculating one-fourth of a 3-days-old PDA plate into a glass jar containing 150 g sterile rice grain:rice hull (RG:RH, 1:3) substrate for 7 days.

*Meloidogyne graminicola*, isolated from infected Asian rice roots from Tarlac and Batangas, Philippines, were maintained on roots of the susceptible Asian rice variety UPLRi-5 at the International Rice Research Institute (IRRI), Los Baños, Philippines. The Tarlac population was used for the phytotron experiment, the Batangas population for the raised bed and greenhouse experiments. Second-stage infective juveniles (J2) were extracted from 3-month-old infected plants by incubation in a mistifier for 48 h (Seinhorst 1962).

### Preparation of plants and soil

Two Asian rice varieties were included in the experiments: the traditional upland variety Palawan (GID 48535, IRRI) and the breeding line IR81413-BB-75-4, which showed respectively synergism and antagonism between the two pathogens in preliminary experiments (Kreye et al. 2010). Seeds were supplied by the Plant Breeding, Genetics and Biotechnology Division of IRRI. Before germination, the seeds were incubated for 5 days at 45 °C to break the dormancy. For the raised bed and greenhouse experiments, seeds were germinated in a layer of fresh water for 7 days at 29/26 °C and a 14/10 h light/dark regime before transplanting. For the phytotron experiment direct seeding was performed.

Soil used in the experiments was taken from the top layer (21 cm) of field B912, part of the experimental farm of IRRI at Los Baños. The soil was a clay loam (45 % silt, 34 % clay and 21 % sand). Natural populations of *M. graminicola* and *Pythium* spp. had been observed in this field before (Banaay et al. 2010). The presence of *M. graminicola* was quantified to 0.35 juveniles per mL soil. Rice variety UPLRi-5 was grown during the season prior to the collection of the soil, to maintain the pathogen populations in the field. Prior to the experiment, weeds were collected from field B912 to verify the presence of both pathogens in the soil. Typical hook-shaped root galls were observed on the root tips of the weeds. The presence of *P. arrhenomanes* was confirmed by cutting discolored roots in 1-cm-pieces and surface-sterilized in 5 % hypochlorite for 1 min. Afterwards they were blotted dry and plated on PDA supplemented with 200 mg/L streptomycin. Hyphae emerging from the roots were transferred to fresh plates and grown for identification. Identification was done by PCR according to Van Buyten & Höfte (2013) with *P. arrhenomanes* (PT60) specific primers in the ITS region (Forward 5’-ATTCTGTACGCGTGGTCTTCCG-3’; Reverse 5’-ACCTCACATCTGCCATCTCTCTCC-3’). This pre-experiment analysis confirmed the natural presence of both pathogens in field B912.

Fertilizer was applied in three parts during the experiments: at 14 days after germination (DAG), 30-35 DAG (at mid-tillering) and 45-50 DAG (at panicle initiation). For the phytotron experiment N, P, K, Zn and Fe at 120:60:40:20:20 kg/ha were applied, whereas for the raised bed and greenhouse experiment N, P, K was applied at 120:40:40 kg/ha final concentration. Plants were watered daily to maintain water tension at field capacity (-10 to -30 kPa at a depth of 15 cm).

### Phytotron experiment set-up

Soil of field B912 was steam-sterilized for 8 h at 100 °C. In this experiment, the interaction of *P. arrhenomanes* and *M. graminicola* on IR81413-BB-75-4 was examined using chopped UPLRi-5 root pieces infected with *M. graminicola* as a nematode inoculum. Infected roots were cut in 1-cm-pieces and 5 g (determined to be equivalent to 1,000 J2) was mixed per kg soil in the upper 1/3 portion of the soil in the PVC pots. Seven day old *P. arrhenomanes* grain:rice hull mixture was incorporated into sterile potting soil at a ratio of 1:20 (inoculum:soil). The same amount of sterile un-inoculated soil was added to the control plants.

After pathogen inoculation in the soil, seeds were directly sown in the pots. Two seeds each were planted at 2-3 cm depth in four equally spaced points in 6-kgs-capacity cylindrical PVC pots. Treatments were as follows: (i) un-inoculated control, (ii) inoculation with *P. arrhenomanes*, (iii) inoculation with *M. graminicola*, and (iv) inoculation with *P. arrhenomanes* + *M. graminicola*. Three replicates (1 pot = 1 replicate) per treatment were prepared and set-up at the phytotron growth chamber. The pots were arranged in a randomized complete block design (RCBD). The phytotron was set at 29 °C/26 °C day/night cycle.

Plant height was assessed at regular time points during the first 60 days after germination (DAG). At harvest the grain weight was evaluated per replicate of all treatments.

### Raised bed experiment set-up

Six adjacent concrete raised beds (each 6.66 m long, 1.05 m wide and 0.21 m deep) were filled with 1500 kg soil from field B912. The raised beds were used to examine three treatments: (i) un-inoculated, steam-sterilized B912 soil as a control treatment, (ii) natural B912 infestation with both *P. arrhenomanes* and *M. graminicola*, and (iii) natural B912 infestation with both pathogens plus additional artificial inoculation with *P. arrhenomanes*. For the artificial *Pythium* inoculation, 15 kg of 7-days-old rice grain:rice hull (RG:RH, 1:3) was mixed in 1,500 kg soil. As a result, treatment (iii) has a higher *P. arrhenomanes* pressure (i.e., additional artificial inoculation with *P. arrhenomanes* PT60), than treatment (ii) (i.e., natural infestation only). The seedlings were spaced 15 cm apart in the raised beds.

Plants were sampled at 10-days-interval during the dry season. Six to eight plants of each genotype and treatment were carefully uprooted and washed under running tap water. Plant growth traits and fresh root weight were determined per plant. Afterwards, roots were cut in 5-mm-pieces and 0.5-1 g of fresh roots was collected for *Pythium* quantification, the rest was used for nematode extraction. The collected root tissue for *Pythium* quantification, consisting of six roots, was split in two biological replicates (except at time points 2, 10 & 20 DAG, where the limited material allowed to sample only 1 replicate) and was directly frozen in liquid nitrogen to preserve the DNA. DNA was extracted using the DNeasy Plant Mini Kit (Qiagen). The quality and concentration of the extracted DNA were determined with a Nanodrop 2000 spectrophotometer (Thermoscientific). Quantitive PCR was performed with three technical replicates for each biological replicate. Primers specific for the internal transcribed spacer (ITS) region of the ribosomal DNA (rDNA) of P. *arrhenomanes* PT60 and primers for plant DNA (reference gene LOC_Os07g02340) were used (Ji et al. 2015; Van Buyten and Höfte 2013). Pure *P. arrhenomanes* DNA and non-infected plant DNA were used to make standard curves.

At 20 days after transplanting, 12 plants were collected to study the nematode development. Root galling and nematode development were assayed after visualization with acid fuchsin staining. Staining was performed by boiling the roots for 3 min in 0.8 % acetic acid and 0.013 % acid fuchsin, washing under running tap water and destaining in 5:100 mL acidified glycerol. At harvest, 12 plants were collected to determine the plant growth traits and yield from the different treatments.

### Greenhouse experiment set-up

Soil of field B912 was steam sterilized for 8 h at 100 °C. Cylindrical PVC pots with a 9 L capacity were filled with 6 kg sieved and sterilized soil from field B912. There were six treatments: (i) un-inoculated control; (ii) *M. graminicola* inoculation; (iii) *P. arrhenomanes* inoculation; (iv) *P. arrhenomanes* + *M. graminicola*; (v) *M. graminicola* + *P. arrhenomanes* 6 days later; and (vi) *P. arrhenomanes* + *M. graminicola* 5 days later. One day prior to transplanting, 150 g of RG:RH was mixed per pot (1:40 ratio) for the *P. arrhenomanes* inoculations. The same amount of sterile un-inoculated soil was added to the other pots. Seven-days-old seedlings were transplanted in four equally spaced points per pot. Soil was watered to near saturation prior to seeding. For the *M. graminicola* inoculation, 6,000 J2 per pot were inoculated with 750 J2 on each side of the seedlings 1 day after transplanting (DAT). The same inoculation procedures were followed for the *P. arrhenomanes*-*M. graminicola* combination treatments.

At two time points, 15 and 20 days after *M. graminicola* inoculation, 12 plants were collected to study the nematode development. At 54 days after *M. graminicola* inoculation, J2 were extracted to determine the reproduction rate for two treatments: (ii) *M. graminicola* single inoculated and (iv) *P. arrhenomanes* + *M. graminicola* inoculated. At harvest, 12 plants were collected to determine the plant growth traits and yield.

### Statistical analysis

Analyses were performed using SPSS v21 software (IBM, USA). Data were statistically analyzed by analysis of variance (ANOVA) and Duncan test (α = 0.05), when the assumptions of normal distribution and homogeneity of variances were met. Not normally distributed data was log(x + 1)-transformed to meet the assumptions for ANOVA or analyzed with Mann-Whitney non-parametric tests (α = 0.05).

## Additional files

Additional file 1: Figure S1. Preliminary phytotron data. (A) Mean plant height of IR81413-BB-75-4 after different treatments over a period of 54 days after germination (*n* = 12). Statistics were performed with Mann-Whitney *U* test (α = 0.05), different letters indicate significant differences. (B) Filled grain weight of IR81413-BB-75-4 plants per pot at harvest grown in soil infested with *M. graminicola* (chopped roots) and *P. arrhenomanes* alone and in combination (*n* = 3). Statistics were performed with One-way ANOVA Duncan test (α = 0.05), different letters indicate significant differences per time point. Error bars are the standard error. (PPTX 99 kb) Additional file 2: Figure S3.
*Pythium arrhenomanes* DNA in rice roots expressed as picogram *Pythium* DNA per nanogram total DNA. Varieties Palawan (A) and IR81413-BB-75-4 (B) quantified with *P. arrhenomanes* specific and plant specific primers at 2, 10, 20, 45 and 60 days after transplanting in the raised bed experiment. ‘Natural infestation’ = soil taken from field B912 and ‘Natural infestation + *P. arrhenomanes’* = B912 soil with additional *P. arrhenomanes* inoculation. Each treatment has two biological replicates (of three pooled plants), except for time points 2, 10 & 20 which consist of one biological replicate (of six pooled plants). (C) *Pythium arrhenomanes* DNA quantification in rice roots from the greenhouse experiment at 12 days after transplanting of three biological replicates, each consisting of 6 pooled plants, that were either *P. arrhenomanes* single infected or *P. arrhenomanes* + *M. graminicola* double infected. Statistics were performed with One-way ANOVA Duncan test (α = 0.05), different letters indicate significant differences. Error bars are the standard error. (PPTX 97 kb) Additional file 3: Figure S2. Nematode development at 20 DAI from greenhouse experiment for Palawan (A,C,E) and IR81413-BB-75-4 (B,D,F) under different infection schemes. (A,B) Total number of nematodes, (C,D) number of galls, and (E,F) the developmental stages of *M. graminicola* per plant at 20 days after transplanting. Statistics were performed with Mann-Whitney *U* test (α = 0.05), different letters indicate significant differences (n = 12). Statistics for (E,F) were performed by giving each group a total score; where the percentage of each stage has a value; J2 = 1; J3/J4 = 2; Females = 3; and Females with Egg masses = 4. Error bars are the standard error. (PPTX 146 kb)

## References

[CR1] Atkinson NJ, Urwin PE (2012). The interaction of plant biotic and abiotic stresses: from genes to the field. J Exp Bot.

[CR2] Bais HP, Weir TL, Perry LG, Gilroy S, Vivanco JM (2006) The role of root exudates in rhizosphere interations with plants and other organisms. In: Annual Review of Plant Biology, vol 57. Annual Review of Plant Biology. Annual Reviews, Palo Alto, pp 233-266. doi:10.1146/annurev.arplant.57.032905.105159 10.1146/annurev.arplant.57.032905.105159 16669762

[CR3] Banaay G, De Waele D, Cuevasa V, Vera Cruz C (2010). Occurrence and distribution of root-infecting pathogens in an aerobic rice field and their association with observed disease symptoms.

[CR4] Bond JP, McGawley EC, Hoy JW (2004). Sugarcane growth as influenced by nematodes and *Pythium arrhenomanes*. Nematropica.

[CR5] Bouman BAM, Peng S, Castañeda AR, Visperas RM (2005). Yield and water use of irrigated tropical aerobic rice systems. Agric Water Manag.

[CR6] Bridge J, Plowright RA, Peng D, Luc M, Sikora RA, Bridge J (2005). Nematode parasites of rice. Plant parasitic nematodes in subtropical and tropical agriculture.

[CR7] Chun SC, Schneider RW (1998). Sites of infection by Pythium species in rice seedlings and effects of plant age and water depth on disease development. Phytopathology.

[CR8] De Waele D, Elsen A (2007). Challenges in tropical plant nematology. Annu. Rev. Physiol..

[CR9] De Waele D, Das K, Zhao D, Tiwari RKS, Shrivastava DK, Vera-Cruz C, Kumar A (2013). Host response of rice genotypes to the rice root-knot nematode (Meloidogyne graminicola) under aerobic soil conditions. Arch Phytopathology Plant Protect.

[CR10] Fernandez L, Cabasan MTN, De Waele D (2013) Life cycle of the rice root-knot nematode *Meloidogyne graminicola* at different temperatures under non-flooded and flooded conditions. Archives Of Phytopathology And Plant Protection:1-8. doi:10.1080/03235408.2013.829627

[CR11] Fiers M, Lognay G, Fauconnier ML, Jijakli MH (2013). Volatile Compound-Mediated Interactions between Barley and Pathogenic Fungi in the Soil. PLoS One.

[CR12] George T, Magbanua R, Garrity DP, Tubaña BS, Quiton J (2002). Rapid yield loss of rice cropped successively in aerobic soil. Agron J.

[CR13] Gheysen G, Mitchum MG (2011). How nematodes manipulate plant development pathways for infection. Curr. Opin. Plant Biol..

[CR14] Griffin GD, Elgin JH (1977). Penetration and development of meloidogyne hapla in resistant and susceptible alfalfa under differing temperatures. J Nematol.

[CR15] Halpin JE, Hanson EW (1958). Effect of age of seedlings of alfalfa, red clover, ladino white clover, and sweet clover on susceptibility to *Pythium*. Phytopathology.

[CR16] Ji H, Kyndt T, He W, Vanholme B, Gheysen G (2014). β-aminobutyric acid–induced resistance against root-knot nematode in rice is based on increased basal defence. Mol. Plant Microbe Interact..

[CR17] Ji H, Gheysen G, Ullah C, Verbeek R, Shang C, De Vleesschauwer D, Höfte M, Kyndt T (2015). The role of thionins in rice defence against root pathogens. Mol. Plant Pathol..

[CR18] Johnson AW, Littrell RH (1970). Pathogenicity of *Pythium aphanidermatum* to *Chrysanthemum* in Combined Inoculations with *Belonolaimus longicaudatus* or *Meloidogyne incognita*. J Nematol.

[CR19] Khan MR, Haque Z (2013). Morphological and biochemical responses of five tobacco cultivars to simultaneous infection with *Pythium aphanidermatum* and *Meloidogyne incognita*. Phytopathol. Mediterr..

[CR20] Kreye C, Bouman BAM, Faronilo JE, Llorca L (2009). Causes for soil sickness affecting early plant growth in aerobic rice. Field Crop Res.

[CR21] Kreye C, Bouman BAM, Reversat G, Fernandez L, Vera Cruz C, Elazegui F, Faronilo JE, Llorca L (2009). Biotic and abiotic causes of yield failure in tropical aerobic rice. Field Crop Res.

[CR22] Kreye C, Das K, Pinili MS, Banaay CG, Elazegui FA, Steelandt A, De Bruyne L, Van Buyten E, Höfte M, De Waele D, Fernandez LC, Llorca L, Faronillo J, Vera Cruz CM, Kumar A, Bouman B (2010). 4th ADB Annual Review.

[CR23] Kyndt T, Fernandez D, Gheysen G (2014). Plant-parasitic nematode infections in rice: molecular and cellular insights. Annu. Rev. Phytopathol..

[CR24] Lafitte HR, Courtois B, Arraudeau M (2002). Genetic improvement of rice in aerobic systems: progress from yield to genes. Field Crops Res.

[CR25] Martin FN, Loper JE (1999). Soilborne plant diseases caused by *Pythium* spp: Ecology, epidemiology, and prospects for biological control. Crit. Rev. Plant Sci..

[CR26] Melendez PL, Powell NT (1967). Histological aspects of fusarium wilt-root knot complex in flue-cured tobacco. Phytopathology.

[CR27] Minton NA, Minton EB (1963). Infection relationship between *Meloidogyne incognita acrita* and *Fusarium oxysporum f. sp. vasinfectum* in cotton. Phytopathology.

[CR28] Padgham JL, Duxbury JM, Mazid AM, Abawi GS, Hossain M (2004). Yield loss caused by *Meloidogyne graminicola* on lowland rainfed rice in Bangladesh. J Nematol.

[CR29] Peng S, Bouman B, Visperas RM, Castañeda A, Nie L, Park HK (2006). Comparison between aerobic and flooded rice in the tropics: Agronomic performance in an eight-season experiment. Field Crops Res.

[CR30] Plowright R, Bridge J (1990). Effect of *Meloidogyne Graminicola* (Nematoda) on the establishment, growth and yield of rice cv IR36. Nematologica.

[CR31] Sandhu N, Jain S, Kumar A, Mehla BS, Jain R (2013) Genetic variation, linkage mapping of QTL and correlation studies for yield, root, and agronomic traits for aerobic adaptation. BMC Genetics 14. doi:10.1186/1471-2156-14-104 10.1186/1471-2156-14-104 PMC4231467 24168061

[CR32] Seinhorst JW (1962). Modifications of the elutriation method for extracting nematodes from soil. Nematologica.

[CR33] Soriano IR, Reversat G (2003). Management of meloidogyne graminicola and yield of upland rice in South-Luzon, Philippines. Nematology.

[CR34] Tuong TP, Bouman BAM, Kijne JW, Barker R, Molden D (2003). Rice production in water-scarce environments. Water productivity in agriculture: limits and opportunities for improvement.

[CR35] Van Buyten E (2013). Pythium spp. affecting aerobic rice cultivation in the Philippines: characterization, intraspecific variability, virulence strategies and interference with plant defense.

[CR36] Van Buyten E, Höfte M (2013). Pythium species from rice roots differ in virulence, host colonization and nutritional profile. BMC Plant Biol.

[CR37] Van Buyten E, Banaay CGB, Vera Cruz C, Höfte M (2013). Identity and variability of Pythium species associated with yield decline in aerobic rice cultivation in the Philippines. Plant Pathol.

[CR38] Vovlas N, Rapoport HF, Jiménez Díaz RM, Castillo P (2005). Differences in Feeding Sites Induced by Root-Knot Nematodes, *Meloidogyne* spp., in Chickpea. Phytopathology.

[CR39] Win PP, Kyi PP, Maung ZTZ, De Waele D (2013). Population dynamics of *Meloidogyne graminicola* and *Hirschmanniella oryzae* in a double rice-cropping sequence in the lowlands of Myanmar. Nematology.

[CR40] Yadeta KA, J. Thomma BPH (2013). The xylem as battleground for plant hosts and vascular wilt pathogens. Front. Plant Sci.

