# Supplementary figures and images for: Interactions between the oomycete Pythium arrhenomanes and the rice root-knot nematode Meloidogyne graminicola in aerobic Asian rice varieties
Source: Rice (N Y). 2016 Jul 29;9:36. doi: 10.1186/s12284-016-0108-3 (PMC4967063; doi:10.1186/s12284-016-0108-3)

## Slide 1
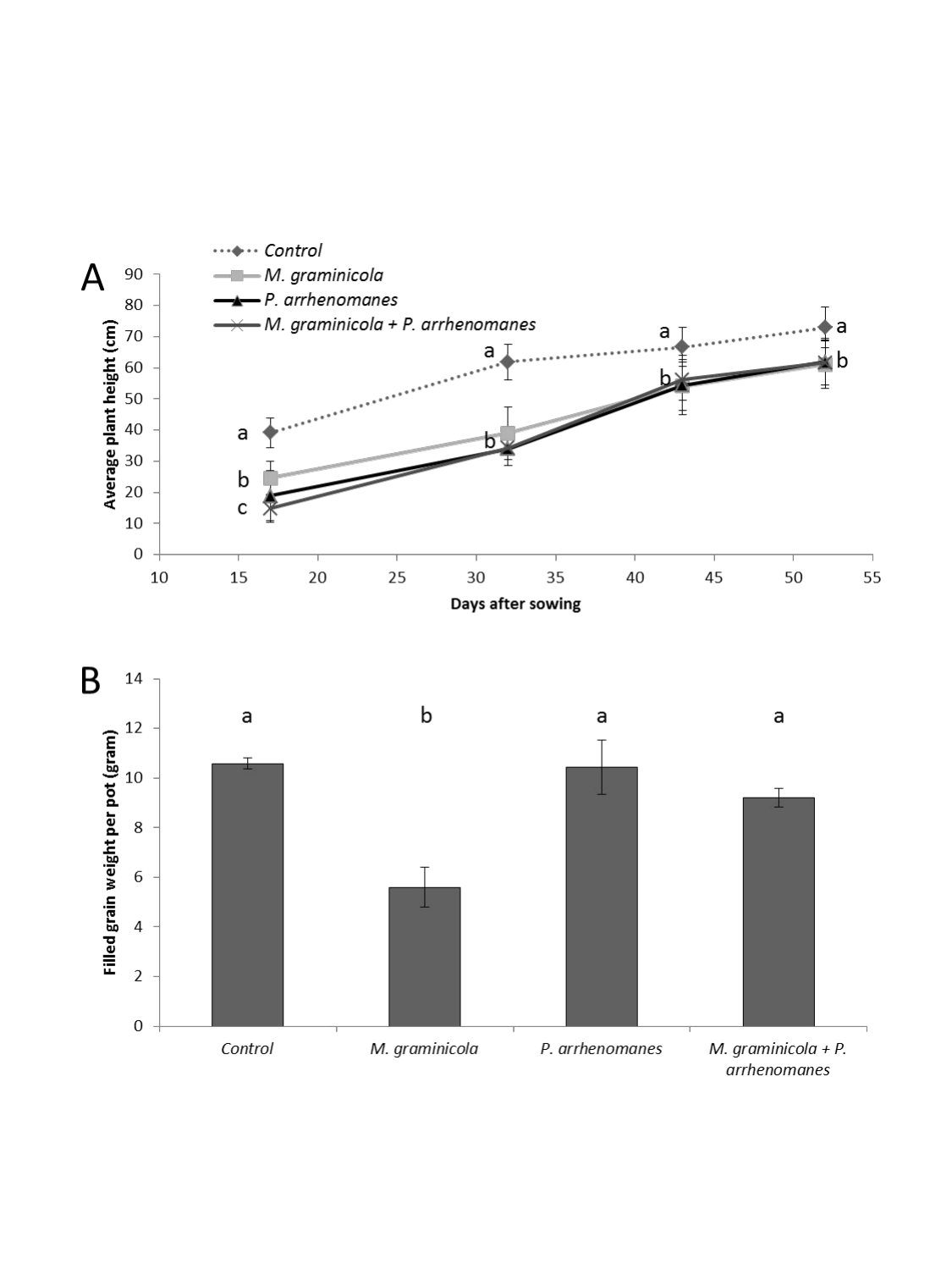

Supplement: Additional file 1: Figure S1. — Preliminary phytotron data. (A) Mean plant height of IR81413-BB-75-4 after different treatments over a period of 54 days after germination (n = 12). Statistics were performed with Mann-Whitney U test (α = 0.05), different letters indicate significant differences. (B) Filled grain weight of IR81413-BB-75-4 plants per pot at harvest grown in soil infested with M. graminicola (chopped roots) and P. arrhenomanes alone and in combination (n = 3). Statistics were performed with One-way ANOVA Duncan test (α = 0.05), different letters indicate significant differences per time point. Error bars are the standard error. (PPTX 99 kb) [file 12284_2016_108_MOESM1_ESM.pptx]

## Slide 1
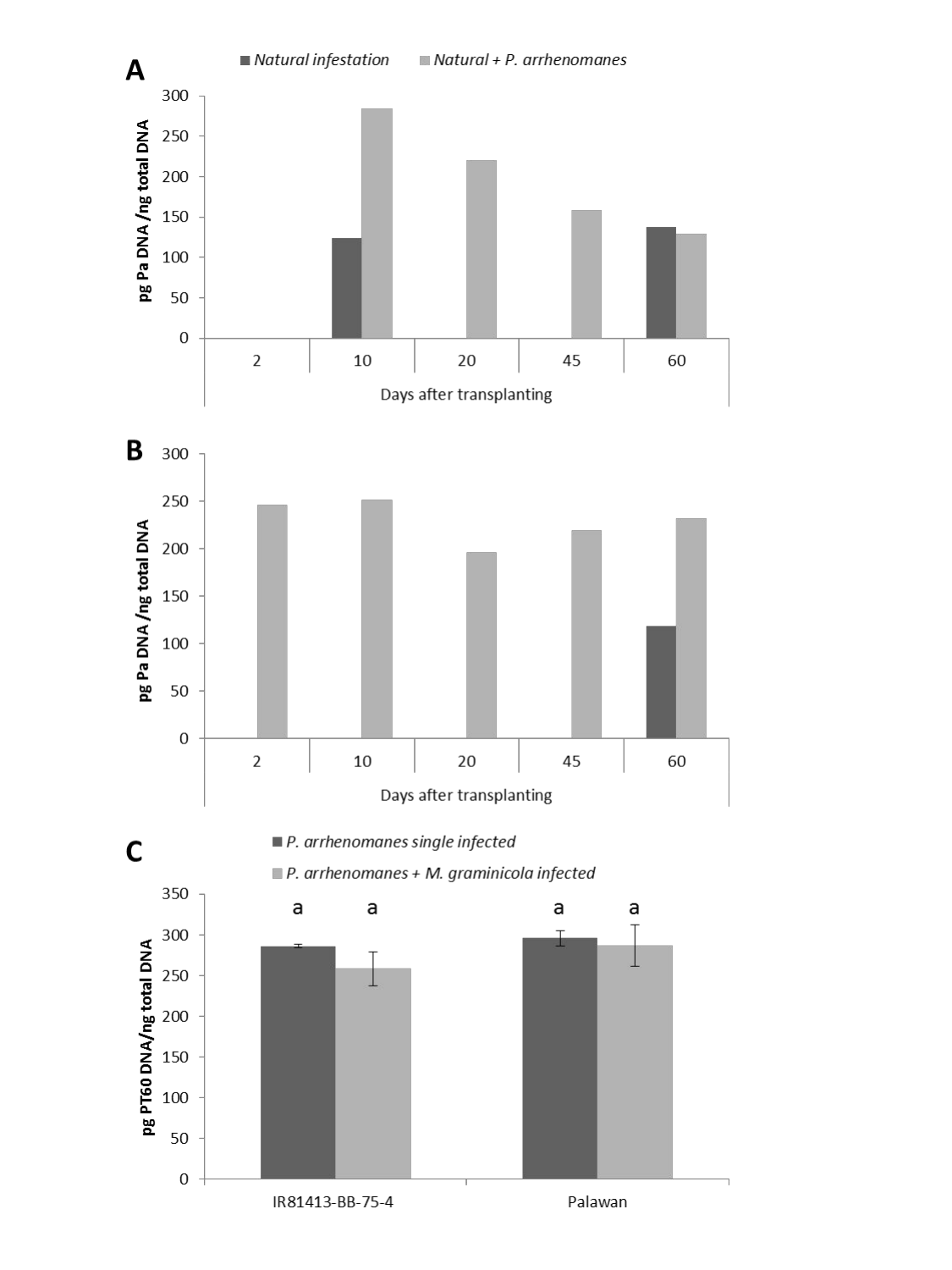

Supplement: Additional file 2: Figure S3. — Pythium arrhenomanes DNA in rice roots expressed as picogram Pythium DNA per nanogram total DNA. Varieties Palawan (A) and IR81413-BB-75-4 (B) quantified with P. arrhenomanes specific and plant specific primers at 2, 10, 20, 45 and 60 days after transplanting in the raised bed experiment. ‘Natural infestation’ = soil taken from field B912 and ‘Natural infestation + P. arrhenomanes’ = B912 soil with additional P. arrhenomanes inoculation. Each treatment has two biological replicates (of three pooled plants), except for time points 2, 10 & 20 which consist of one biological replicate (of six pooled plants). (C) Pythium arrhenomanes DNA quantification in rice roots from the greenhouse experiment at 12 days after transplanting of three biological replicates, each consisting of 6 pooled plants, that were either P. arrhenomanes single infected or P. arrhenomanes + M. graminicola double infected. Statistics were performed with One-way ANOVA Duncan test (α = 0.05), different letters indicate significant differences. Error bars are the standard error. (PPTX 97 kb) [file 12284_2016_108_MOESM2_ESM.pptx]

## Slide 1
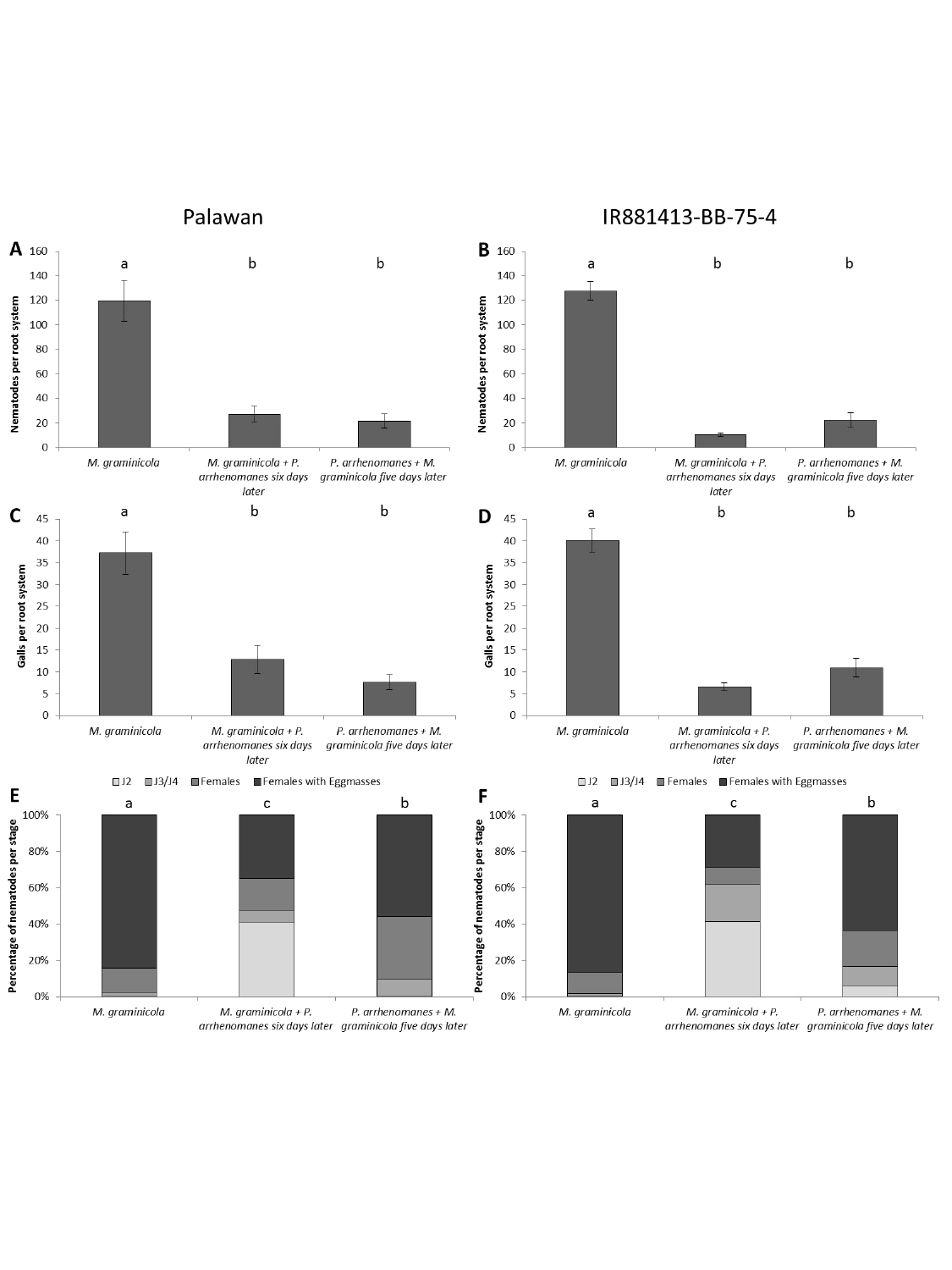

Supplement: Additional file 3: Figure S2. — Nematode development at 20 DAI from greenhouse experiment for Palawan (A,C,E) and IR81413-BB-75-4 (B,D,F) under different infection schemes. (A,B) Total number of nematodes, (C,D) number of galls, and (E,F) the developmental stages of M. graminicola per plant at 20 days after transplanting. Statistics were performed with Mann-Whitney U test (α = 0.05), different letters indicate significant differences (n = 12). Statistics for (E,F) were performed by giving each group a total score; where the percentage of each stage has a value; J2 = 1; J3/J4 = 2; Females = 3; and Females with Egg masses = 4. Error bars are the standard error. (PPTX 146 kb) [file 12284_2016_108_MOESM3_ESM.pptx]
